# Feeding Habits of Narrow-Clawed Crayfish *Pontastacus leptodactylus*: Implications for Stock Enhancement and Aquaculture

**DOI:** 10.3390/ani16142155

**Published:** 2026-07-11

**Authors:** Ying Yan, Ming Li, Yanjie Tang, Xiting Chen, Haibo Jiang, Muzi Zhang, Na Li, Bin Li

**Affiliations:** 1 School of Marine Sciences, Ningbo University, Ningbo 315211, China; 2401130144@nbu.edu.cn (Y.Y.); 2301130097@nbu.edu.cn (Y.T.); 2411130076@nbu.edu.cn (X.C.); 2 College of Animal Science, Guizhou University, Guiyang 550025, China; jhb99412@126.com (H.J.); mzzhang3@gzu.edu.cn (M.Z.); 3 College of Biosystems Engineering and Food Science, Zhejiang University, Hangzhou 310058, China; 4 College of Veterinary Medicine, Xinjiang Agricultural University, Urumqi 830052, China; nali@xjau.edu.cn

**Keywords:** *Pontastacus leptodactylus*, eDNA metabarcoding, fatty acid signature analysis, in vitro/in vivo digestibility, intestinal health

## Abstract

The narrow-clawed crayfish (*Pontastacus leptodactylus*), a non-native species accidentally introduced into China’s Irtysh River Basin, has formed a wild population with aquaculture potential, but its natural diet and protein utilization remain poorly understood. This study investigated its feeding ecology and digestive responses to protein sources to support ecological assessment and feed formulation. Intestinal contents and muscle samples from wild crayfish were analyzed using eDNA metabarcoding and fatty acid signature analysis. In vitro digestibility of 10 protein ingredients was evaluated with crude digestive enzyme extracts, and a 4-week trial compared an all-plant-protein diet (PPD) with an all-animal-protein diet (APD). eDNA indicated an omnivorous diet dominated by planktonic taxa, especially Rotifera, and fatty acid biomarkers suggested contributions from diatoms, dinoflagellates, phytoplankton, zooplankton, and benthic organisms. Soybean meal and soy protein concentrate showed high dry matter digestibility, whereas fishmeal and krill meal had high crude protein digestibility and amino acid release. Compared with PPD, APD increased feed intake, apparent nutrient digestibility, hemolymph nutritional indicators, villus height, and intestinal trypsin and lipase activities; however, intestinal integrity was preserved in both groups, and most intestinal antioxidant indices did not differ significantly. These findings indicate that animal proteins enhance nutrient utilization in *P. leptodactylus*, while selected plant proteins may serve as partial alternatives.

## Simple Summary

The narrow-clawed crayfish is a non-native species that has been accidentally introduced into China’s Irtysh River Basin and may become a valuable species for cold-water farming. However, little is known about what it eats in nature or which feed ingredients it can use well. This study aimed to describe its natural food sources and test how it digests different protein ingredients, so that safer management and better feeds can be developed. We examined food remains and biological signals in wild crayfish and tested several common plant and animal protein sources in the laboratory and in a short feeding trial. The crayfish ate a wide range of foods, mainly small drifting plants and animals in the water, showing that it is an adaptable feeder. Among feed ingredients, soybean meal and soy protein concentrate were useful plant options, while fishmeal and krill meal were more easily digested as protein sources. Compared with the all-plant-protein diet, the all-animal-protein diet improved feed intake, nutrient digestion, blood nutrient indicators, and intestinal digestive activity, while both diets maintained normal gut structure and showed largely similar intestinal antioxidant enzyme activities. These findings can help farmers design more sustainable crayfish feeds and support responsible use and monitoring of this non-native species.

## 1. Introduction

The narrow-clawed crayfish (*Pontastacus leptodactylus*) is a freshwater decapod species of ecological and economic significance, widely distributed across Eastern Europe and parts of the Middle East [1]. It was first recorded in the Irtysh River Basin of Xinjiang, China, and is considered a non-native species accidentally introduced into this region [2]. In the present study, “established” refers to the formation of a wild population that can be repeatedly collected from the Irtysh River Basin, rather than evidence of an intentional stocking program. As a cold-water crayfish species with high market value, it has attracted increasing attention in China, particularly in the context of the rapid expansion of red swamp crayfish aquaculture (*Procambarus clarkii*) [3,4]. Compared with red swamp crayfish, narrow-clawed crayfish exhibits potential advantages in cooler environments and may occupy a differentiated market niche within high-value crayfish production [5]. Recent breakthroughs in artificial breeding, including large-scale juvenile production under controlled conditions, have further improved its aquaculture feasibility.

Aquaculture and stock enhancement represent two principal strategies for increasing aquatic animal production [6]. However, successful stock enhancement and sustainable farming depend on a clear understanding of species-specific feeding ecology [7]. Moreover, this is particularly critical for invasive species, as their trophic interactions can significantly alternative food webs and ecosystem functioning [8]. Despite the formation of a wild population in China’s Irtysh River Basin, information on the natural diet and trophic position of narrow-clawed crayfish in invaded ecosystems remains limited, constraining both ecological risk assessment and feed development.

Multiple techniques have been employed to investigate aquatic animal feeding habits, including microscopic gut content analysis, stable isotope analysis, fatty acid signature analysis, DNA barcoding, and eDNA metabarcoding [9,10]. Among these, eDNA metabarcoding enables high-resolution taxonomic identification of partially digested prey items and is less affected by prey life stage or morphological degradation [11,12]. Fatty acid signature analysis (FASA) complements molecular approaches by reflecting assimilated dietary components through conservative fatty acid transfer from prey to consumer [13]. Specific fatty acids function as trophic biomarkers, such as C16:1n7 and C20:5n3 for diatoms, and C20:1 and C22:1 for herbivorous copepods [14,15,16,17]. The integration of eDNA metabarcoding and fatty acid signature analysis therefore provides a comprehensive framework for elucidating both ingested and assimilated dietary components, allowing for a more accurate reconstruction of feeding ecology [18].

From a nutritional perspective, crustaceans generally require relatively high dietary protein levels to support growth and molting [19]. However, protein ingredients constitute the most expensive component of aquafeeds, significantly affecting production costs [20]. Previous studies suggest that the optimal dietary protein level for narrow-clawed crayfish ranges between 30% and 39%, with limited additional growth benefits at higher inclusion levels [21]. In addition to quantity, protein quality and digestibility are critical determinants of feed efficiency [22]. Fishmeal (FM) has traditionally served as the primary high-quality protein source in crustacean diets [23], yet concerns regarding sustainability, price volatility, and resource limitation have intensified the search for alternative protein sources [20,24]. High-quality alternatives should exhibit balanced amino acid profiles, high digestibility, and low levels of antinutritional factors [25,26]. Nevertheless, the digestibility of commonly used protein sources in narrow-clawed crayfish has not been systematically evaluated, limiting evidence-based feed formulation.

Therefore, this study had two primary objectives: (1) to elucidate the feeding ecology of wild narrow-clawed crayfish through the combined application of eDNA metabarcoding and fatty acid signature analysis, and (2) to determine the in vitro and in vivo digestibility of commonly used protein sources in this species, thereby providing a scientific basis for cost-effective and nutritionally optimized feed formulation. By integrating ecological and nutritional approaches, this research aims to support ecological risk assessment and sustainable aquaculture development of this non-native species.

## 2. Materials and Methods

### 2.1. Ethical Statement

All animal handling procedures were performed in strict accordance with the Guidelines for the Care and Use of Experimental Animals of Ningbo University, and were approved under license number 20190410-042.

### 2.2. Experimental Crayfish and Experimental Design

**Figure 1:**
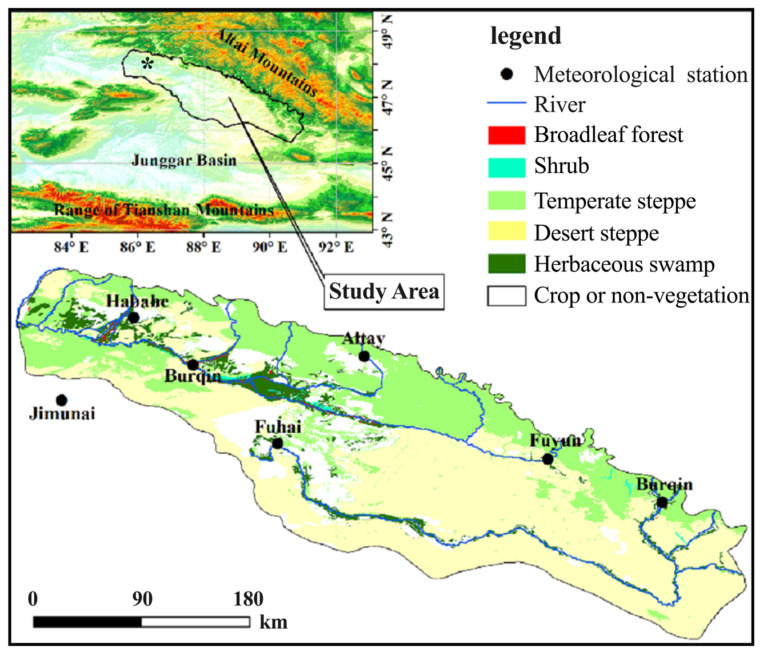
Geographical location map of the Irtysh River basin [27] and the geographical location of the sampling site (*).

A total of 135 wild narrow-clawed crayfish were collected from the lower reaches of the Irtysh River in Xinjiang, China (47°52′08″ N, 86°17′08″ E; Figure 1) on 30 August 2024. 30 similarly sized individuals (mean body weight = 85.16 ± 6.18 g) were selected for intestinal content and muscle tissue sampling for eDNA metabarcoding and fatty acid analyses, with 10 crayfish pooled per sample to yield three replicates for each analysis.

An additional 45 crayfish of uniform size (mean body weight = 64.55 ± 4.58 g) were randomly assigned to three groups (15 individuals per group). Under sterile conditions, intestines and stomachs were dissected, weighed, and homogenized to 10% (*w*/*v*) in ice-cold 0.2 M phosphate buffer (PBS; pH 7.4 for intestines, pH 5.0 for stomachs). The homogenates were centrifuged at 5000× *g* for 20 min at 4 °C, and the supernatants were collected as crude digestive enzyme extracts and stored at −20 °C until analysis.

Another 60 crayfish with an initial body weight of 77.84 ± 4.09 g were randomly assigned to six tanks (70 L each, approximately 780 g biomass per tank, 10 individuals per tank), with three replicates per treatment. Before the formal feeding trial, all crayfish were acclimated for 2 weeks and fed a commercial crayfish diet containing 35% crude protein and 8% crude lipid. After acclimation, crayfish were randomly assigned to two isonitrogenous and isoenergetic dietary treatments: an all-plant-protein diet (PPD) and an all-animal-protein diet (APD). The formal feeding trial lasted 4 weeks under the following culture conditions: temperature, 20 ± 1 °C; dissolved oxygen, >7.5 mg/L; and pH, 7.8 ± 0.4. During the first 2 weeks of the feeding trial, crayfish were adapted to their assigned experimental diets, and feces were collected during the final 2 weeks for apparent digestibility determination. At the end of the feeding trial, crayfish were fasted for 24 h before sampling. Before sampling, surface water was gently removed and body weight was recorded. Two crayfish were randomly selected from each tank, giving six individuals per treatment (*n* = 6 per treatment). The selected crayfish were anesthetized in an ice-water bath for 15 min. Hemolymph (approximately 0.5 mL) was withdrawn from the posterior region of the cephalothorax using a sterile 1 mL syringe and transferred into a 1.5 mL centrifuge tube containing an equal volume of anticoagulant. The samples were kept at 4 °C for 4 h and then centrifuged at 12,000 rpm for 20 min. The supernatant was collected and stored at −80 °C for subsequent physiological and biochemical analyses. After hemolymph collection, the intestine was dissected and transferred into a 2 mL centrifuge tube, rapidly frozen in liquid nitrogen, and stored at −20 °C for enzyme activity assays. A portion of the midgut was fixed in tissue fixative in a 2 mL centrifuge tube for subsequent histological sectioning.

### 2.3. eDNA Metabarcoding Analysis

Genomic DNA was extracted from crayfish intestinal contents using the TIANamp Marine Animals DNA Kit (TIANGEN, Beijing, China). The integrity of the isolated genomic DNA was verified by 1% agarose gel electrophoresis, and the DNA was stored at −20 °C until further use. PCR amplification and high-throughput sequencing were performed by Shanghai BIOZERON Co., Ltd. (Shanghai, China). The V9 region of the 18S rRNA gene was amplified with the primer set 18SV9F (5′-CCCTGCCNTTTGTACACAC-3′) and 18SV9FR (5′-CCTTCNGCAGGTTCACCTAC-3′) [28]. The COI gene was amplified using primers MlCOIintF (5′-GGWACWGGWTGAACWGTWTAYCCYCC-3′) and JghHCO2198 (5′-TAIACYTCIGGRTGICCRAARAAYCA-3′) [29]. PCR products were confirmed by 2% agarose gel electrophoresis, quantified using the QuantiFluor™-ST system (Promega, Madison, WI, USA), and sequenced on an Illumina sequencing platform. High-quality sequences were obtained after quality filtering and chimera removal. Non-redundant sequences (excluding singletons) were clustered into operational taxonomic units (OTUs) at 97% sequence similarity using Uparse v7.1. Representative sequences of each OTU were taxonomically assigned using the RDP classifier to determine community composition and relative abundance [30].

### 2.4. Fatty Acid Signature Analysis

Fatty acid content was determined by Panomix Biomedical Tech Co., Ltd. (Suzhou, China). Muscle samples were methylated with 2 mL of 1% sulfuric acid-methanol. A 500 μL aliquot of the supernatant was mixed with 25 μL methyl salicylate. Fatty acids were quantified by an Agilent 6890N/5975B GC-MS (Agilent Technologies, Santa Clara, CA, USA) fitted with an HP-INNOWAX column (30 m × 0.25 mm). A 1 μL sample was injected in splitless mode. Temperatures of the injector, ion source, transfer line, and quadrupole were 250, 230, 250, and 150 °C, respectively. The oven program was: 50 °C for 3 min; 10 °C/min to 220 °C, hold 3 min; 15 °C/min to 250 °C, hold 10 min. Helium was used as carrier gas at 1.0 mL/min. The characteristic fatty acid profiles were obtained via a systematic review of prior research [31,32,33,34,35,36,37].

### 2.5. In Vitro Digestibility Assay

10 commonly used protein feed ingredients, including both animal- and plant-based sources, were tested: peanut meal (PNM), soybean meal (SBM), rapeseed meal (RSM), corn gluten meal (CGM), soy protein concentrate (SPC), FM, krill meal (KM), fish bone meal (FBM), spray-dried plasma (SDP), and poultry by-product meal (PBM). Samples (0.25 g) were placed into 50 mL tubes, mixed with 7 mL of PBS and 2 mL of crude enzyme extract, and subjected to in vitro digestion at 20 ± 1 °C with shaking at 50 rpm for 6 h. Dry matter digestibility, crude protein digestibility, and amino acid release were measured in triplicate (*n* = 3). Dry matter digestibility = (Initial feed weight − Residue weight)/Initial feed weight × 100; crude protein digestibility = (Initial feed weight × Feed crude protein content − Residue weight × Residue crude protein content)/(Initial feed weight × Feed crude protein content) × 100. Amino acids in the hydrolysates collected at 1 h, 2 h, 4 h and 6 h were quantified using the o-phthaldialdehyde (OPA) method [38].

### 2.6. In Vivo Digestibility Trial

Based on the results of the in vitro digestibility assay, plant and animal protein ingredients with relatively high digestibility were selected to formulate two isonitrogenous and isoenergetic experimental diets: PPD and APD. Yttrium oxide (Y_2_O_3_) was added at 0.1% as an inert marker to determine apparent digestibility coefficients. Diets were pelleted to a diameter of 1.5 mm using a screw-type pelletizer (CD2-1TS, Guangzhou Huagong Optoelectronic Technology Co., Ltd., Guangzhou, China). The pellets were subjected to high-temperature processing and subsequently dried at 60 °C. The dried diets were stored at −20 °C until use. Detailed diet formulation and proximate composition are presented in Table 1.

### 2.7. Biochemical Analysis

Proximate composition (moisture, crude ash, crude protein, and crude lipid) of the feeds was determined according to the method described by [24]. Briefly, moisture content was measured by oven-drying samples at 105 °C to a constant weight; crude ash was determined by incinerating dried samples in a muffle furnace at 550 °C for 5 h; crude protein was analyzed via the Kjeldahl method (N × 6.25); and crude lipid was extracted with petroleum ether using a Soxhlet extractor. Y_2_O_3_ contents in feeds and feces were determined by Guobiao (Beijing) Testing & Certification Co., Ltd. (Beijing, China).

Hemolymph biochemical parameters [total protein (TP), glucose (GLU), triglycerides (TG), total cholesterol (T-CHO), alanine aminotransferase (ALT), aspartate aminotransferase (AST)] were quantified using commercial kits from Nanjing Jiancheng Bioengineering Institute (Nanjing, China). Similarly, intestinal antioxidant enzyme activities [malondialdehyde (MDA), total antioxidant capacity (T-AOC), reduced glutathione (GSH), catalase (CAT), superoxide dismutase (SOD)] and digestive enzyme activities [α-amylase (AMS), trypsin (TPS), lipase (LPS)] were determined using standardized kits from the same manufacturer.

### 2.8. Intestinal Histopathology

Intestinal tissues were fixed, dehydrated in graded ethanol, cleared in xylene, and paraffin-embedded; 5 μm sections were cut with a rotary microtome (YD-202A, Jinhua YIDI Medical Appliance Co., Ltd., Jinhua, China) and H&E-stained using reagents from Nanjing Jiancheng Bioengineering Institute (D006-1-1). Sections were observed and photographed under a Nikon TS100 microscope (Nikon, Tokyo, Japan). Qualitative histological evaluation of the intestine focused on villus height (VH) and villus width (VW) measured using ImageJ (version 1.53; National Institutes of Health, Bethesda, MD, USA), as well as intestinal structural integrity.

### 2.9. Statistical Analyses

Data from comparisons between the two dietary treatments were analyzed using independent-sample *t*-tests. For comparisons among the ten feed ingredients in the in vitro digestibility assay, one-way ANOVA followed by Tukey’s multiple comparison test was used. Prior to statistical analysis, data normality was assessed using the Shapiro–Wilk test, and homogeneity of variances was evaluated using Levene’s test. All datasets met the assumptions of normality and homogeneity of variance (*p* > 0.05). All continuous variables are presented as mean ± standard error of the mean (SEM), and statistical significance was defined as *p* < 0.05. All analyses were conducted using IBM SPSS Statistics version 27.0 (IBM Corp., Armonk, NY, USA). Spearman’s rank correlation analysis and graphical visualization were performed using GraphPad Prism version 10.3.1 (GraphPad Software, Inc., La Jolla, CA, USA).

## 3. Results

### 3.1. Taxonomic Composition in the Intestinal Contents

**Figure 2:**
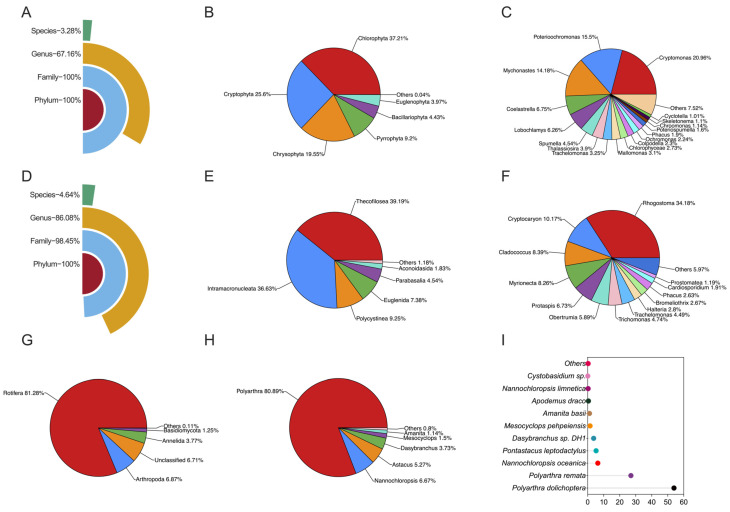
Taxonomic composition in the intestinal contents (*n* = 3). (**A**): Phytoplankton annotation ratios at different taxonomic levels. (**B**): Phytoplankton composition at the phylum level. (**C**): Phytoplankton composition at the genus level. (**D**): Zooplankton annotation ratios at different taxonomic levels. (**E**): Zooplankton composition at the phylum level. (**F**): Zooplankton composition at the genus level. (**G**): Eukaryotic composition at the phylum level. (**H**): Eukaryotic composition at the genus level. (**I**): Eukaryotic composition at the species level.

The taxonomic composition of phytoplankton (Figure 2A–C), zooplankton (Figure 2D–F) and eukaryotes (Figure 2G–I) in the intestinal contents, as determined by eDNA metabarcoding, is presented in Figure 2. Phytoplankton taxonomic annotation achieved complete assignment at the phylum and family levels (100%), whereas lower annotation rates were observed at the genus (67.16%) and species (3.80%) levels (Figure 2A). At the phylum level (Figure 2B), the community was dominated by Chlorophyta (37.21%), Cryptophyta (25.60%), and Chrysophyta (19.55%). Less abundant phyla included Pyrrophyta (9.20%), Bacillariophyta (4.43%), and Euglenophyta (3.97%), while taxa classified as “others” collectively accounted for only 0.04% of the total relative abundance. At the genus level (Figure 2C), *Cryptomonas* (20.96%), *Poterioochromonas* (15.50%), and *Mychonastes* (14.18%) were the predominant genera, whereas the remaining genera exhibited comparatively low relative abundances.

Zooplankton taxonomic assignment reached 100% at the phylum level, 98.45% at the family level, and 86.08% at the genus level, whereas only 4.64% of sequences were assigned at the species level (Figure 2D). At the phylum level (Figure 2E), the zooplankton community was mainly composed of Thecofilosea (39.19%) and Intramacronucleata (36.63%). Additional taxa included Polycystinea (9.25%), Euglenida (7.38%), Parabasalia (4.54%), and Aconoidasida (1.83%), while the “others” category accounted for 1.18% of the total relative abundance. At the genus level (Figure 2F), *Rhogostoma* (34.18%) was the most abundant genus, followed by *Cryptocaryon* (10.17%) and *Cladococcus* (8.39%), whereas the remaining genera were detected at comparatively low relative abundances.

For eukaryotes, as shown in Figure 2G, Rotifera was the dominant phylum, accounting for 81.28% of the total relative abundance, followed by Arthropoda (6.87%) and Annelida (3.77%). The “unclassified” and “other” categories comprised 6.71% and 0.11% of the community, respectively. At the genus level (Figure 2H), *Polyarthra* was the predominant genus (80.89%), whereas the remaining genera were detected at comparatively low abundances, including *Nannochloropsis* (6.67%), *Astacus* (5.27%), and *Dasybranchus* (3.73%). At the species level (Figure 2I), *Polyarthra dolichoptera* (53.90%) and *Polyarthra remata* (26.99%) were the dominant species, followed by *Nannochloropsis oceanica* (6.35%) and *Pontastacus leptodactylus* (5.27%), each representing a substantially lower proportion of the community.

### 3.2. Muscle Fatty Acid Composition

A total of 49 fatty acids were identified in this study (Table 2), comprising 16 saturated fatty acids (SFAs) and 33 unsaturated fatty acids (UFAs). SFAs accounted for 27.82% of total fatty acids, whereas UFAs represented 72.18%, including 19 monounsaturated fatty acids (MUFAs, 27.62%) and 14 polyunsaturated fatty acids (PUFAs, 44.56%). Eleven fatty acids exhibited relative abundances exceeding 1%, including two SFAs (C16:0 and C18:0), three MUFAs (C16:1, C18:1n9c, and C18:1n7), and six PUFAs (C18:2n6, C18:3n3, C20:2, C20:4n6, C20:5n3, and C22:6n3). Overall, the total UFAs content was approximately 2.6-fold higher than that of SFAs. Among all detected fatty acids, C20:5n3 was the predominant component, accounting for 25.71% of total fatty acids. Other major fatty acids included C16:0 (18.88%) and C18:1n9c (14.94%).

Characteristic fatty acids can be used to indicate the feeding habits of organisms. The results showed that 14 main characteristic fatty acids were detected in the muscle of crayfish, namely C15:0, C16:1t, C16:1, C17:0, C18:1n9t, C18:1n9c, C18:1n7, C18:2n6, C18:2n6t, C20:1, C18:3n3, C20:4n6, C20:5n3, and C22:6n3. Among them, C20:5n3, C18:1n9c, C18:2n6, C18:1n7, and C20:4n6 were present at relatively high levels. Based on the characteristic fatty acid profiles, the main food sources of crayfish included diatoms, dinoflagellates, brown algae, zooplankton, phytoplankton, and benthic organisms.

### 3.3. Correlation Analysis

**Figure 3:**
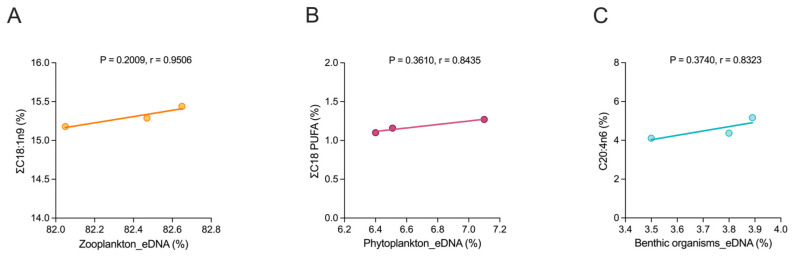
Spearman correlations between dietary composition inferred from eDNA metabarcoding and fatty acid biomarkers in crayfish muscle (*n* = 3). (**A**): Relationship between zooplankton-derived eDNA abundance and zooplankton-associated fatty acids (ΣC18:1n9). (**B**): Relationship between phytoplankton-derived eDNA abundance and plant-associated fatty acids (ΣC18 PUFA). (**C**): Relationship between benthic prey-derived eDNA abundance and arachidonic acid (C20:4n6).

Spearman correlation analysis was conducted to examine the relationships between dietary composition inferred from eDNA metabarcoding and corresponding fatty acid biomarkers in crayfish muscle (Figure 3). A strong positive association was observed between the relative abundance of zooplankton-derived eDNA and the proportion of zooplankton-associated fatty acids (ΣC18:1n9) (R = 0.95, *P* = 0.20; Figure 3A). Similarly, the relative abundance of phytoplankton-derived eDNA was positively correlated with phytoplankton-associated fatty acids (ΣC18 PUFA) (R = 0.84, *P* = 0.36; Figure 3B), and the relative abundance of benthic organisms-derived eDNA was positively correlated with C20:4n6 (R = 0.83, *P* = 0.37; Figure 3C). None of these relationships reached statistical significance, which is likely attributable to the limited sample size. Collectively, these correlations suggest consistent variation trends between dietary components identified by eDNA metabarcoding and nutrient assimilation patterns reflected by fatty acid profiles.

### 3.4. In Vitro Digestibility of Dietary Protein Ingredients

**Figure 4:**
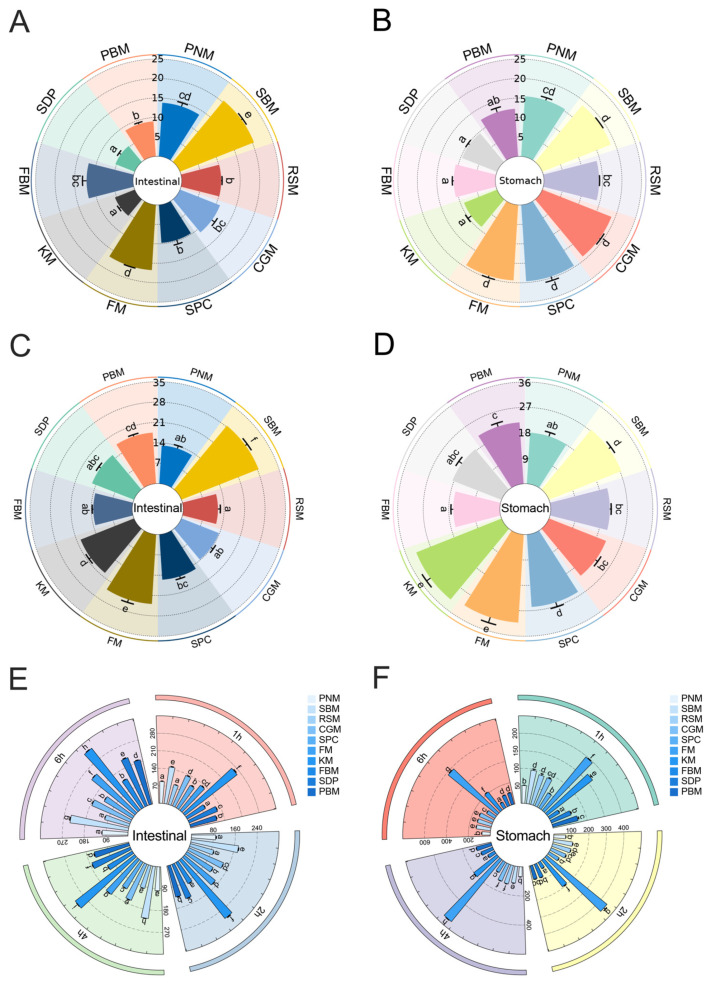
Dry matter digestibility, crude protein digestibility, and amino acid release of ten common feed ingredients using crude enzyme extracts from the intestine and stomach (*n* = 3). (**A**) Dry matter digestibility using intestinal crude enzymes; (**B**) dry matter digestibility using stomach crude enzymes; (**C**) crude protein digestibility using intestinal crude enzymes; (**D**) crude protein digestibility using stomach crude enzymes; (**E**) amino acid release using intestinal crude enzymes; (**F**) amino acid release using stomach crude enzymes. PNM = peanut meal, SBM = soybean meal, RSM = rapeseed meal, CGM = corn gluten meal, SPC = soy protein concentrate, FM = fishmeal, KM = krill meal, FBM = fish bone meal, SDP = spray-dried plasma, and PBM = poultry by-product meal. Data were expressed as the mean ± SEM. Significant differences among groups (*p* < 0.05) were indicated by different letters on the diagrams.

As shown in Figure 4A, in the intestinal enzyme system, SBM exhibited significantly higher dry matter digestibility than all other ingredients (*p* < 0.05). Among the animal derived ingredients, FM showed significantly greater dry matter digestibility than the remaining treatments (*p* < 0.05). In the gastric enzyme system (Figure 4B), SPC, CGM, and SBM displayed relatively high dry matter digestibility among the plant protein sources, whereas FM achieved the highest digestibility among the animal protein ingredients, followed by PBM. A similar pattern was observed for crude protein digestibility. In the intestinal enzyme system, SBM and FM exhibited significantly higher crude protein digestibility than the other treatments (*p* < 0.05; Figure 4C). In the gastric enzyme system, FM and KM showed the highest crude protein digestibility among all ingredients (*p* < 0.05; Figure 4D). Regarding amino acid release, KM demonstrated the highest release efficiency in the intestinal enzyme system, significantly exceeding that of all other ingredients (*p* < 0.05; Figure 4E). Among the plant protein sources, SBM exhibited significantly greater amino acid release efficiency than the other plant proteins (*p* < 0.05; Figure 4E). In the gastric enzyme system, FM and KM showed significantly higher amino acid release efficiency than the remaining protein ingredients (*p* < 0.05; Figure 4F).

### 3.5. Growth Performance and Apparent Nutrient Digestibility

As shown in Table 3, no mortality occurred in either treatment group during the four-week culture period, and the survival rate was 100% in both groups. There were no significant differences in FBW, WGR, SGR or FCR between the two treatment groups (*p* > 0.05). Compared with the PPD treatment group, the APD treatment group exhibited a significant increase in feed intake (*p* < 0.05). In addition, the ADC_DM_ (*p* < 0.05), ADC_CP_ (*p* < 0.05), ADC_CL_ (*p* < 0.05) and ADC_Ash_ (*p* < 0.05) values in the APD treatment group were significantly higher than those in the PPD treatment group.

### 3.6. Hemolymph Biochemistry

**Figure 5:**
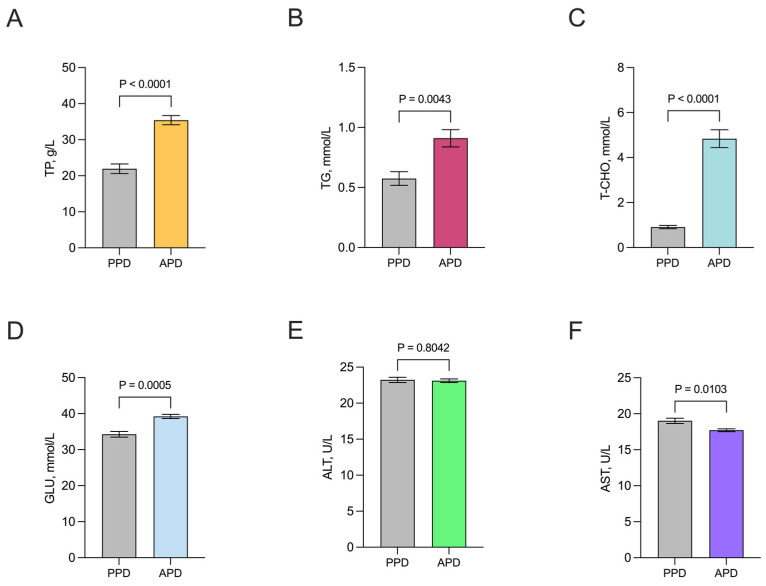
Effects of different protein-source diets on the hemolymph biochemical parameters of crayfish (*n* = 6). (**A**): TP = Total protein. (**B**): TG = Triglyceride. (**C**): T-CHO = Total cholesterol. (**D**): GLU = Glucose. (**E**): ALT = Alanine aminotransferase. (**F**): AST = Aspartate aminotransferase. All data presented as means ± SEM.

As shown in Figure 5, the hemolymph contents of TP (*p* < 0.05), TG (*p* < 0.05), T-CHO (*p* < 0.05), and GLU (*p* < 0.05) were significantly higher in the APD group than in the PPD group, whereas AST activity (*p* < 0.05) was significantly lower. No significant difference in ALT activity was observed between the two groups (*p* > 0.05).

### 3.7. Intestinal Histology

**Figure 6:**
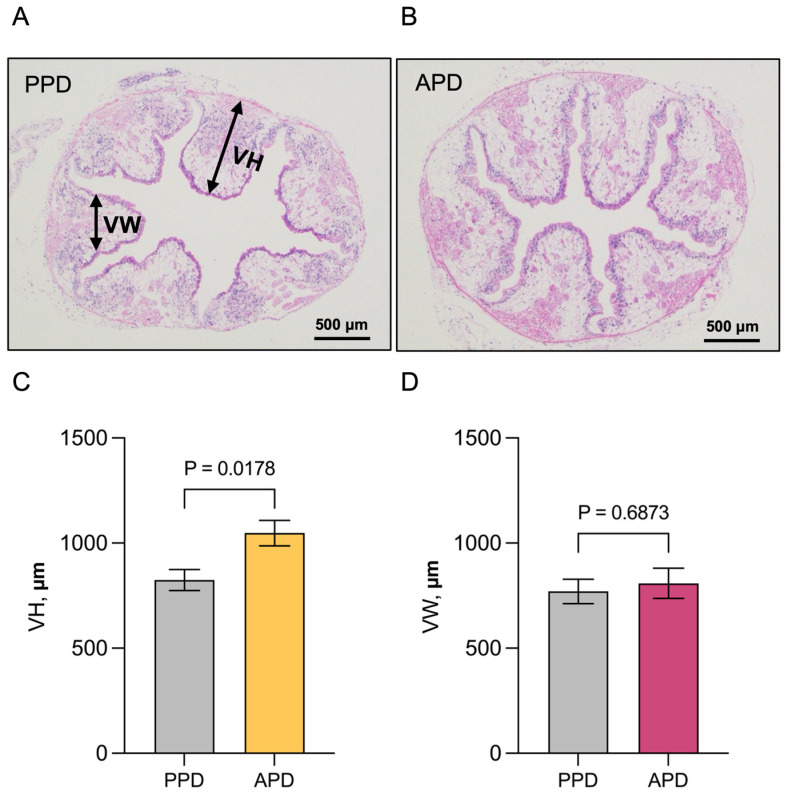
Effects of different protein-source diets on the intestinal histological structure of crayfish (*n* = 6). (**A**): Intestinal section of crayfish in the PPD group. (**B**): Intestinal section of crayfish in the APD group. (**C**): VH of crayfish. (**D**): VW of crayfish. All data presented as means ± SEM.

Histological examination revealed well-preserved intestinal morphology in both groups (Figure 6A,B). The intestinal villi were intact, regularly arranged, and structurally clear, with no evidence of epithelial damage, inflammatory infiltration, or mucosal disruption. Further statistical analysis of VH (Figure 6C) and VW (Figure 6D) showed that the APD treatment group exhibited significantly greater VH than the PPD treatment group (*p* < 0.05), whereas no significant difference in VW was observed between the two groups (*p* > 0.05).

### 3.8. Intestinal Digestive Enzyme Activities

**Figure 7:**
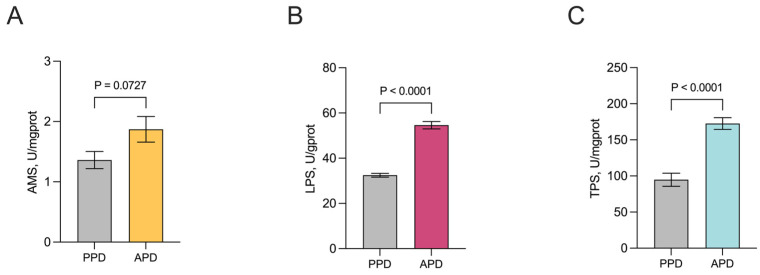
Effects of different protein-source diets on intestinal digestive enzyme activities in crayfish (*n* = 6). (**A**): AMS = α-amylase. (**B**): LPS = Lipase. (**C**): TPS = Trypsin. All data presented as means ± SEM.

Intestinal digestive enzyme activities of crayfish in the two treatment groups are presented in Figure 7. The activities of LPS (Figure 7B) and TPS (Figure 7C) in the APD group were significantly higher than those in the PPD group (*p* < 0.05). In contrast, no significant differences in AMS (Figure 7A) activity were observed between the groups (*p* > 0.05).

### 3.9. Intestinal Antioxidant Indices

**Figure 8:**
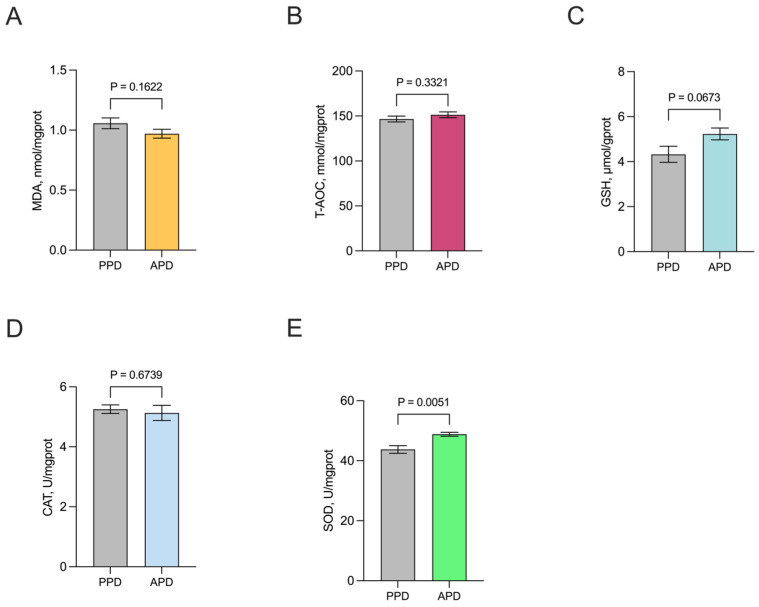
Effects of different protein-source diets on intestinal antioxidant enzyme activities in crayfish (*n* = 6). (**A**): MDA = Malondialdehyde. (**B**): T-AOC = Total antioxidant capacity. (**C**): GSH = Glutathione. (**D**): CAT = Catalase. (**E**): SOD = Superoxide dismutase. All data presented as means ± SEM.

There were no significant differences in intestinal MDA content, T-AOC activity, GSH content, or CAT activity between the two treatment groups (*p* > 0.05; Figure 8A–D). However, the SOD activity in the APD treatment group was significantly higher than that in the PPD treatment group (*p* < 0.05; Figure 8E).

## 4. Discussion

As a non-native, accidentally introduced, cold-water, and economically valuable species in China, the feeding ecology of the narrow-clawed crayfish provides a critical basis for ecological risk assessment and stock enhancement [40]. In this study, eDNA metabarcoding results revealed a diet primarily composed of phytoplankton (Chlorophyta, Cryptophyta, and Chrysophyta) and zooplankton (Thecofilosea and Intramacronucleata). Notably, Rotifera dominated the eukaryotic community, with *Polyarthra dolichoptera* and *Polyarthra remata* identified as the most prevalent species. This trophic profile reinforces the well-documented omnivorous feeding strategy of freshwater crayfish, which opportunistically exploit diverse plant and animal resources across multiple trophic levels [21,41,42,43]. The prevalence of plankton-derived taxa in the gut likely mirrors the trophic structure of the Irtysh River basin [44]. This ecosystem is characterized by highly diverse zooplankton communities dominated by rotifers and cladocerans, alongside phytoplankton assemblages primarily consisting of Chlorophyta, Bacillariophyta, and Cryptophyta [44,45]. Our findings suggest that this species efficiently exploits these abundant planktonic resources, a trait that likely facilitates its successful establishment and expansion in invaded freshwater ecosystems. Furthermore, the detection of conspecific DNA in intestinal contents is noteworthy. Although eDNA metabarcoding cannot distinguish between active cannibalism, scavenging, or incidental ingestion, conspecific DNA suggests that intraspecific consumption may occur under natural conditions. Cannibalistic behavior is widely reported in freshwater crayfish and is commonly associated with high population density, food limitation, habitat disturbance, or vulnerability during the molting cycle [46]. Such trophic flexibility may enhance survival and population persistence under fluctuating environmental conditions and may therefore contribute to the successful establishment of the narrow-clawed crayfish in invaded ecosystems. From an ecological perspective, cannibalism may influence population regulation and community interactions, whereas in aquaculture systems it may increase mortality and affect stocking-density management [47]. Therefore, further studies are needed to determine the frequency and ecological significance of cannibalism in wild populations and its implications for invasion dynamics and aquaculture production.

Fatty acid signature analysis serves as a robust methodology for determining the trophic ecology of crustaceans, given the stable transfer of fatty acids across food webs [48,49]. In this study, 49 fatty acids were identified, UFAs predominating at 72.18% of the total. Notably, C20:5n3 (EPA) was the most prevalent, acting as a vital essential fatty acid for growth, ecdysis, cell membrane fluidity, and cold-water adaptation in crustaceans [50,51,52]. Given the limited capacity for *de novo* synthesis of long-chain polyunsaturated fatty acids, these findings underscore the necessity of supplementing commercial diets with EPA-rich lipids to ensure optimal physiological performance [53,54]. Furthermore, the detection of 14 specific trophic markers—including indicators for diatoms [31,33], zooplankton [36], phytoplankton [34,35], and benthic organisms [39]—provides biochemical confirmation of an omnivorous diet consistent with our eDNA metabarcoding results.

Moreover, the Spearman correlation analysis revealed positive associations between the relative abundance of eukaryotic taxa identified by eDNA metabarcoding and their corresponding fatty acid biomarkers. Although none of these correlations reached statistical significance, the overall concordance between the molecular and biochemical datasets may suggest that the two approaches provide complementary information for characterizing the diet of narrow-clawed crayfish. However, these results should be interpreted with caution because the correlation analysis was based on a limited number of pooled biological replicates (*n* = 3), which substantially reduced statistical power. Therefore, the present correlations should be regarded as exploratory rather than confirmatory, and future studies with larger sample sizes are needed to rigorously evaluate the relationship between dietary composition and fatty acid assimilation. Our results nevertheless indicate that crayfish exploit a diverse resource base comprising phytoplankton, zooplankton, and benthic organisms, facilitating their successful establishment and competitive dominance in cold-water ecosystems [8,55]. Because the dietary analysis was based on one sampling site and one sampling date, and because pooled biological replicates were used, the feeding ecology results should be interpreted as a site- and season-specific baseline rather than a full population-level characterization. Future studies should include larger sample sizes, multiple habitats, and seasonal sampling.

Digestibility serves as a fundamental proxy for the bioavailability of nutrients and the physiological capacity of aquatic species to utilize specific feed ingredients [56,57]. By simulating species-specific digestive physiology, the in vitro assay uses gastrointestinal enzyme extracts to catalyze feed hydrolysis ex vivo, enabling rapid and high-resolution evaluation of the digestive and absorptive capacity of aquatic animals for individual dietary components [58,59,60]. Given the high protein demands of crustaceans for growth and reproduction [19,61], this study evaluated the in vitro dry matter and crude protein digestibility, along with amino acid release efficiency, of 10 representative protein sources. Our results showed that, within the intestinal crude enzyme system, SBM exhibited the highest in vitro dry matter digestibility, followed by FM and PNM. In the gastric enzyme system, SPC showed the highest in vitro dry matter digestibility, followed by FM, CGM, and SBM. These results suggest that the crayfish generally exhibits higher digestive efficiency for dry matter from plant-derived protein sources than from animal derived sources. This pattern is consistent with previous findings reported in other crustacean species, including traditional tiger shrimp (*Penaeus monodon*) [62] and Pacific white shrimp (*Litopenaeus vannamei*) [63]. The observed differences may be partly attributed to the relatively high ash content commonly present in animal-derived protein ingredients, which may limit dry matter solubilization by acting as a physical buffer within the digestion matrix [64]. Moreover, KM, FM, SBM, and SPC exhibited relatively higher levels of in vitro protein digestibility. This finding is consistent with previous reports in Brazilian pink shrimp (*Farfantepenaeus paulensis*) and Pacific white shrimp [65]. FM is widely recognized as a high-quality animal protein source, and its digestibility and utilization in shrimp diets are generally superior to those of most other feed ingredients [66]. KM is another important animal derived protein source [67]. Previous studies have shown that replacing 26.7–40.0% of FM with KM significantly improves the growth performance and feed utilization efficiency of oriental river prawn [67]. SBM, characterized by a balanced amino acid profile, low cost, and stable supply, is one of the most widely used plant protein sources in shrimp feeds [68]. Bae et al. [69] reported that replacing 20% or 40% of FM with SBM did not significantly affect the growth performance or lysozyme activity of juvenile Pacific white shrimp. SPC, a processed product derived from SBM contains over 65% protein and reduced levels of anti-nutritional factors such as trypsin inhibitors and saponins [70]. It has been reported that complete replacement of FM with SPC in Pacific white shrimp diets using fish oil as the lipid source does not adversely affect the growth [71]. Moreover, the results of in vitro simulated amino acid production were consistent with these findings: amino acid release was relatively high in KM, SBM, and FM. After ingestion, proteins must be digested into amino acids to be absorbed by animals [72]. Therefore, proteins that are easily hydrolyzed can be more efficiently utilized by aquatic animals [73]. In conclusion, when formulating compound feeds for the crayfish, the inclusion levels of KM, SBM, and SPC may be appropriately increased.

Based on in vitro results, APD and PPD were formulated to evaluate their effects on in vivo nutrient digestibility and physiological status of crayfish. While survival reached 100% in both groups, the APD group exhibited significantly higher ADC_DM_, ADC_CP_, ADC_CL_, and ADC_Ash_. These results indicate a superior capacity of crayfish to utilize animal-derived nutrients, consistent with findings in other decapods such as palaemonid shrimp (*Macrobrachium americanum*) [74] and Pacific white shrimp [75,76]. The higher feed intake observed in the APD group suggests enhanced palatability, likely due to the abundance of free amino acids and nucleotides in FM or KM, which act as potent chemoattractants for crustaceans [77,78]. Furthermore, elevated hemolymph levels of TP, TG, T-CHO, and GLU in the APD group reflect a robust nutritional and metabolic status, as high-quality protein typically correlates with enhanced energy metabolism [79,80,81]. Notably, the significantly higher AST activity in the PPD group, despite stable ALT levels, may hint at mild hepatopancreatic stress or impairment induced by an all-plant diet, a phenomenon warranting further histological investigation [82].

Intestinal health is a primary determinant of nutrient utilization and physiological status in aquatic animals, typically evaluated through morphology, digestive enzyme activities, and antioxidant defense systems [83,84]. In the present study, histological observations revealed that both diets maintained structural integrity, with no evidence of epithelial damage or inflammatory cell infiltration, suggesting that the PPD did not induce overt intestinal injury. Similar findings have been reported in Pacific white shrimp, in which high levels of fishmeal replacement by fermented soybean meal or cottonseed protein concentrate did not induce obvious intestinal histopathological alterations or inflammatory responses [85,86]. Despite the lack of pathology, VH was significantly higher in the APD group compared to the PPD group, whereas VW remained unaffected. As VH is positively correlated with the absorptive surface area, the increased VH in the APD group likely enhances the contact between nutrients and epithelial cells, thereby improving absorption efficiency [87]. This morphological enhancement may represent a physiological adaptation to the higher nutrient density and superior protein quality of animal-derived diets [88]. These observations align with previous studies demonstrating that high-quality protein sources can optimize intestinal architecture—specifically increasing VH and absorptive capacity—to ultimately improve feed utilization efficiency in aquatic species [80,89,90].

Digestive enzyme activity is a direct indicator of nutrient processing and utilization efficiency in aquatic animals [91]. A similar inhibitory trend has been reported in Redclaw crayfish (*Cherax quadricarinatus*), where diets containing single plant protein sources significantly reduced trypsin and pepsin activities compared with a fishmeal-based control diet [92]. In the present study, trypsin and lipase activities were significantly lower in the PPD group compared to the APD group. This reduction is likely attributable to the presence of anti-nutritional factors inherent in plant-based ingredients, such as protease inhibitors and tannins, which can directly inhibit enzyme secretion or form complexes that impair nutrient hydrolysis and absorption [66,93].

Intestinal antioxidant capacity is another important indicator of intestinal health, as oxidative stress can compromise barrier function and impair nutrient absorption [94,95]. In this study, no significant differences were observed between the APD and PPD groups regarding intestinal MDA content, GSH content, T-AOC activity, or CAT activity. This stability indicates that the all-plant protein diet did not induce oxidative stress or lipid peroxidation in the crayfish intestine [96]. Overall, these findings suggest that while a PPD does not cause overt pathological damage or oxidative stress, it significantly limits apparent nutrient digestibility and feed intake. Consequently, although plant proteins such as SBM are viable candidates for partially replacing FM in crayfish formulations, the inclusion of phagostimulants or functional additives may be useful for enhancing palatability and stimulating digestive enzyme secretion, thereby improving feed utilization in sustainable aquaculture. Taken together, the ecological and physiological datasets provide complementary evidence for the trophic flexibility of the narrow-clawed crayfish. The eDNA metabarcoding and fatty acid results indicate that wild crayfish exploit a broad dietary spectrum, including phytoplankton, zooplankton, and benthic organisms, whereas the digestibility and feeding-trial results demonstrate that this species can physiologically utilize both plant- and animal-derived protein sources. Nevertheless, the superior feed intake, nutrient digestibility, hemolymph nutritional status, intestinal fold development, and digestive enzyme activities observed in the APD group indicate that animal proteins were more efficiently utilized under the present experimental conditions. Thus, the integration of ecological diet reconstruction and physiological feeding responses suggests that the successful establishment of this species may be partly associated with broad trophic flexibility, while also providing guidance for future feed formulation studies. It should also be noted that the present feeding trial compared only two extreme dietary treatments, namely an all-plant-protein diet and an all-animal-protein diet. While this design allowed a clear comparison of the physiological responses to contrasting protein sources, it does not permit the determination of optimal plant-protein inclusion or fishmeal replacement levels for practical feed formulation. Therefore, the present findings should be regarded as a preliminary evaluation of protein-source utilization, and future studies incorporating graded substitution levels, longer feeding durations, and comprehensive growth-performance assessments are required to establish practical and sustainable feed formulation strategies for narrow-clawed crayfish aquaculture.

## 5. Conclusions

This study integrated eDNA metabarcoding, fatty acid signature analysis, in vitro digestibility assays, and a feeding trial to characterize the feeding ecology and protein utilization of the narrow-clawed crayfish. The results demonstrated that this species is an omnivorous feeder whose natural diet is dominated by planktonic organisms, particularly rotifers, phytoplankton, and zooplankton. Fatty acid biomarkers further indicated dietary contributions from diatoms, dinoflagellates, phytoplankton, zooplankton, and benthic organisms. Among the tested feed ingredients, soybean meal and soy protein concentrate showed the highest digestibility among plant protein sources, whereas fishmeal and krill meal exhibited the greatest crude protein digestibility and amino acid release among animal protein sources. In the feeding trial, the all-animal-protein diet produced the best overall nutritional responses, including higher feed intake, nutrient digestibility, hemolymph nutritional indicators, villus height, and digestive enzyme activities, whereas the all-plant-protein diet resulted in comparatively lower digestive and absorptive performance. Nevertheless, both diets maintained normal intestinal morphology and similar antioxidant status, indicating that selected plant proteins can be utilized without causing obvious intestinal damage. Collectively, these findings provide the first integrated assessment of feeding ecology and dietary protein utilization in the non-native Chinese population of narrow-clawed crayfish in the Irtysh River Basin, offering a scientific foundation for ecological risk assessment, stock enhancement programs, and the development of cost-effective and sustainable aquaculture feeds.

## Figures and Tables

**Table 1:** Formulation and nutritional composition of experimental diets.

Items	PPD	APD
Feed ingredients (%, dry matter)		
Soybean meal	15.00	0.00
Corn gluten meal	25.00	0.00
Soy protein concentrate	15.00	0.00
Fishmeal	0.00	25.00
Krill meal	0.00	15.00
Poultry by-product meal	0.00	10.00
High-gluten wheat flour	24.00	26.00
Soybean oil	4.00	4.00
Phospholipid oil	5.00	3.00
Vitamin premix ^1^	1.00	1.00
Mineral premix ^2^	1.00	1.00
Monocalcium phosphate	2.00	2.00
Lysine	0.70	0.00
Methionine	0.20	0.15
Threonine	0.30	0.30
Choline chloride	0.50	0.50
Salt	1.00	1.00
Vitamin C ester	0.02	0.02
Chitinase	0.20	0.20
Microcrystalline cellulose	3.48	9.23
Zeolite powder	1.50	1.50
Y_2_O_3_	0.10	0.10
Total	100.00	100.00
Proximate composition, % diet ^3^		
Moisture	2.74 ± 0.19	2.28 ± 0.13
Crude protein	35.92 ± 0.56	36.10 ± 0.34
Crude lipid	13.25 ± 0.18	13.98 ± 0.21
Ash	8.20 ± 0.28	14.60 ± 0.05
NFE ^4^	39.89	33.04
GE (kJ/g diet) ^5^	20.58	19.73

^1^ Mixed vitamin (g/kg): Inositol 25 g, Choline 100 g, Vitamin B_12_ 5 g, Niacin 25 g, Vitamin B_6_ 5 g, Folic acid 1 g, Vitamin B_2_ 5 g, Biotin 0.25 g, Vitamin B_1_ 5 g, Vitamin C 10 g, Vitamin K_3_ 1 g, Vitamin A 0.15 g, Vitamin E 2.5 g, Pantothenic acid 10 g, Vitamin D_3_ 0.00125 g. ^2^ Mixed minerals (g/kg): NaSeO_3_ 0.02 g, MgSO_4_·7H_2_O 147.4 g, (NH_4_)_2_MoS_4_ 0.06 g, CoCl_2_·6H_2_O 0.08 g, NaCl 49.8 g, KI 0.16 g, C_12_H_22_FeO_14_ 10.9 g, CuSO_4_·5H_2_O 0.62 g, ZnSO_4_·7H_2_O 4.67 g, MnSO_4_·H_2_O 3.12 g. ^3^ Except for NFE and GE, all values were measured. NFE and GE were calculated as described below. All data presented as means ± SEM (*n* = 3). ^4^ NFE = nitrogen-free extract. NFE (%) = 100 − moisture (%) − crude protein (%) − crude lipid (%) − ash (%). ^5^ GE = gross energy. GE (kJ/g diet) = [crude protein (%) × 23.6 + crude lipid (%) × 39.5 + NFE (%) × 17.2]/100.

**Table 2:** Fatty acid composition of muscle tissue (*n* = 3).

Fatty Acid	Common Name	Content (μg/g)	Percentage (%)	Trophic Indicator	References
C6:0	Caproic acid	0.1390 ± 0.0136	0.0167 ± 0.0014		
C8:0	Caprylic acid	0.0251 ± 0.0102	0.0030 ± 0.0012		
C10:0	Capric acid	0.0251 ± 0.0070	0.0030 ± 0.0008		
C11:0	Undecylic acid	0.0350 ± 0.0031	0.0042 ± 0.0003		
C12:0	Lauric acid	0.2690 ± 0.0302	0.0322 ± 0.0032		
C13:0	Tridecylic acid	0.1133 ± 0.0036	0.0136 ± 0.0004		
C14:0	Myristic acid	2.3339 ± 0.0667	0.2804 ± 0.0070		
C14:1t	trans-Myristoleic acid	0.4687 ± 0.1639	0.0558 ± 0.0187		
C14:1	Myristoleic acid	0.6775 ± 0.0318	0.0813 ± 0.0024		
C15:0	Pentadecylic acid	3.8510 ± 0.1894	0.4622 ± 0.0146	Planktonic bacteria	[32]
C15:1t	trans-Pentadecenoic acid	0.2804 ± 0.0237	0.0338 ± 0.0032		
C15:1	Pentadecenoic acid	0.9342 ± 0.0969	0.1120 ± 0.0106		
C16:0	Palmitic acid	157.1786 ± 3.9727	18.8810 ± 0.2326		
C16:1t	trans-Palmitoleic acid	2.3240 ± 0.1052	0.2789 ± 0.0079	Diatoms	
C16:1	Palmitoleic acid	26.7489 ± 1.6314	3.2212 ± 0.241	Diatoms	[33]
C17:0	Margaric acid	7.2739 ± 0.3219	0.8732 ± 0.0253	Planktonic bacteria	
C17:1t	trans-Heptadecenoic acid	2.2240 ± 0.0500	0.2672 ± 0.003		[31]
C17:1	Heptadecenoic acid	6.1160 ± 0.3939	0.7339 ± 0.0366		
C18:0	Stearic acid	54.6276 ± 3.1720	6.5541 ± 0.2677		[32]
C18:1n9t	Elaidic acid	2.9973 ± 0.2034	0.3595 ± 0.0182	Brown algae/zooplankton	
C18:1n7t	trans-Vaccenic acid	1.1633 ± 0.1602	0.1392 ± 0.0168		
C18:1n12	Petroselinic acid	8.2737 ± 1.4202	0.9987 ± 0.1851		
C18:1n9c	Oleic acid	124.3668 ± 2.7039	14.9401 ± 0.058	Brown algae/zooplankton	
C18:1n7	Vaccenic acid	41.8406 ± 1.0392	5.0262 ± 0.0588	Planktonic bacteria	[36]
C18:2n6t	Linolelaidic acid	0.8539 ± 0.0921	0.1023 ± 0.0093	Phytoplankton	
C19:1n9t	trans-Nonadecenoic acid	0.5038 ± 0.0202	0.0605 ± 0.0015		
C18:2n6	Linoleic acid	50.5753 ± 1.2769	6.0833 ± 0.2288	Phytoplankton	
C20:0	Arachidic acid	2.2884 ± 0.1019	0.2747 ± 0.0074		[36]
C18:3n6	gamma-Linolenic acid	3.4960 ± 0.0301	0.4202 ± 0.0054		
C20:1t	trans-Eicosenoic acid	0.7617 ± 0.0844	0.0912 ± 0.0086		[37]
C20:1	Eicosenoic acid	6.2694 ± 0.1225	0.7536 ± 0.0170	Herbivorous copepods	
C18:3n3	alpha-Linolenic acid	8.9178 ± 0.5211	1.0729 ± 0.0706	Phytoplankton	[34]
C21:0	Heneicosylic acid	1.2704 ± 0.0294	0.1526 ± 0.0008		
C20:2	Eicosadienoic acid	11.4182 ± 0.2746	1.3715 ± 0.0094		
C22:0	Behenic acid	0.4732 ± 0.0897	0.0565 ± 0.0098		[35]
C20:3n6	Dihomo-gamma-linolenic acid	1.2888 ± 0.0822	0.1546 ± 0.0072		
C22:1n9t	Brassidic acid	0.6399 ± 0.0991	0.0765 ± 0.0106		
C22:1n9	Erucic acid	2.4059 ± 0.5276	0.2872 ± 0.0584		
C20:3n3	Eicosatrienoic acid	2.7440 ± 0.0970	0.3297 ± 0.0104		
C20:4n6(ARA)	Arachidonic acid	37.9458 ± 3.2845	4.5488 ± 0.3186	Benthic organisms	[39]
C23:0	Tricosylic acid	1.1536 ± 0.0126	0.1387 ± 0.0017		
C22:2	Docosadienoic acid	1.0662 ± 0.2200	0.1272 ± 0.0244		[34]
C20:5n3(EPA)	Eicosapentaenoic acid	213.9081 ± 0.8668	25.7135 ± 0.3778	Diatoms/cryptophytes	
C24:0	Lignoceric acid	0.5838 ± 0.0251	0.0701 ± 0.0023		
C24:1	Tetracosenoic acid	0.8725 ± 0.1567	0.1042 ± 0.0170		
C22:4	Docosatetraenoic acid	0.6217 ± 0.0841	0.0745 ± 0.0094		
C22:5n6	Docosapentaenoic acid n-6	1.5040 ± 0.0864	0.1804 ± 0.0073		
C22:5n3	Docosapentaenoic acid n-3	3.9115 ± 0.1118	0.4707 ± 0.0216		
C22:6n3(DHA)	Docosahexaenoic acid	32.5651 ± 0.4576	3.9131 ± 0.0165	Dinophyceae	
TFA	Total fatty acids	832.3259 ± 15.0036	100.0000 ± 0.0000		
SFA	Saturated fatty acids	231.6409 ± 7.4862	27.8162 ± 0.4132		
MUFA	Monounsaturated fatty acids	229.8686 ± 3.5845	27.6209 ± 0.1592		
PUFA	Polyunsaturated fatty acids	370.8164 ± 4.1625	44.5629 ± 0.3069		

All data presented as means ± SEM (*n* = 3).

**Table 3:** Effects of different protein-source diets on the growth performance and apparent nutrient digestibility of crayfish (*n* = 3).

Items	PPD	APD	*p*
IBW, g/crayfish	77.30 ± 5.16	78.38 ± 6.86	0.902
FBW, g/crayfish	81.81 ± 5.10	84.18 ± 7.06	0.791
WGR, %	5.98 ± 0.52	7.57 ± 0.84	0.140
SGR, %/day	0.21 ± 0.02	0.26 ± 0.03	0.138
FI, g/crayfish	6.75 ± 0.03	7.17 ± 0.12	0.006
FCR	1.51 ± 0.07	1.29 ± 0.11	0.105
SR, %	100.00 ± 0.00	100.00 ± 0.00	-
ADC_DM_, %	76.43 ± 0.48	84.00 ± 0.58	<0.001
ADC_CP_, %	96.93 ± 0.06	98.14 ± 0.05	<0.001
ADC_CL_, %	91.75 ± 0.26	94.52 ± 0.22	0.001
ADC_Ash_, %	93.56 ± 0.19	97.40 ± 0.07	<0.001

IBW = Initial body weight, FBW = final body weight, WGR = weight gain rate, SGR = specific growth rate, FI = feed intake, FCR = feed conversion ratio, SR = survival rate, ADC_DM_ = Apparent dry matter digestibility, ADC_CP_ = Apparent protein digestibility, ADC_CL_ = Apparent lipid digestibility, and ADC_Ash_ = Apparent ash digestibility. All data presented as means ± SEM (*n* = 3).

## Data Availability

The data presented in this study are available from the corresponding author upon reasonable request. The data are not publicly available because they form part of an ongoing research project.
